# Iron Retention in Root Hemicelluloses Causes Genotypic Variability in the Tolerance to Iron Deficiency-Induced Chlorosis in Maize

**DOI:** 10.3389/fpls.2018.00557

**Published:** 2018-04-26

**Authors:** Rongli Shi, Michael Melzer, Shaojian Zheng, Andreas Benke, Benjamin Stich, Nicolaus von Wirén

**Affiliations:** ^1^Department of Physiology and Cell Biology, Leibniz-Institute for Plant Genetics and Crop Plant Research, Gatersleben, Germany; ^2^State Key Laboratory of Plant Physiology and Biochemistry, College of Life Sciences, Zhejiang University, Hangzhou, China; ^3^Max Planck Institute for Plant Breeding Research, Cologne, Germany

**Keywords:** cell wall, apoplastic iron, iron–phosphorus interaction, Fe deficiency, strategy II

## Abstract

Antagonistic interactions of phosphorus (P) hamper iron (Fe) acquisition by plants and can cause Fe deficiency-induced chlorosis. To determine the physiological processes underlying adverse Fe–P interactions, the maize lines B73 and Mo17, which differ in chlorosis susceptibility, were grown hydroponically at different Fe:P ratios. In the presence of P, Mo17 became more chlorotic than B73. The higher sensitivity of Mo17 to Fe deficiency was not related to Fe–P interactions in leaves but to lower Fe translocation to shoots, which coincided with a larger pool of Fe being fixed in the root apoplast of P-supplied Mo17 plants. Fractionating cell wall components from roots showed that most of the cell wall-contained P accumulated in pectin, whereas most of the Fe was bound to root hemicelluloses, revealing that co-precipitation of Fe and P in the apoplast was not responsible for Fe inactivation in roots. A negative correlation between chlorophyll index and hemicellulose-bound Fe in 85 inbred lines of the intermated maize B73 × Mo17 (IBM) population indicated that apoplastic Fe retention contributes to genotypic differences in chlorosis susceptibility of maize grown under low Fe supplies. Our study indicates that Fe retention in the hemicellulose fraction of roots is an important determinant in the tolerance to Fe deficiency-induced chlorosis of graminaceous plant species with low phytosiderophore release, like maize.

## Introduction

Among common crop species, graminaceous species are considered as relatively tolerant to Fe deficiency-induced chlorosis, which is based on the fact that Fe-deficient graminaceous plants synthesize and release mugineic acid-type phytosiderophores for the subsequent uptake of Fe(III)-phytosiderophores (Römheld and Marschner, 1986a). In contrast to dicots, which acquire Fe mainly via Fe(III) reduction, phytosiderophore-mediated Fe(III) chelation is only a little hampered by increasing soil pH (Römheld and Marschner, 1986b). Thus, graminaceous crops are usually able to efficiently acquire Fe also in calcareous or high pH soils where Fe availability is poor. Previous studies have shown that there is a large species-dependent variation in particular regarding phytosiderophore release, with species like barley and oat releasing larger amounts of phytosiderophores than species like rice or maize (Kawai et al., 1988; Römheld and Marschner, 1990). This lower capacity of phytosiderophore synthesis and release may cause yield losses when plants are grown on soils with low Fe availability. While transgenic approaches increasing phytosiderophore biosynthesis were proven efficient in enhancing Fe acquisition (Takahashi et al., 2001; Kobayashi and Nishizawa, 2012), conventional breeding approaches exploiting the existing genotypic variation in phytosiderophore release are hampered by rapid microbial degradation of phytosiderophores (von Wirén et al., 1994, 1995) and have remained rare or restricted to a small number of genotypes (Clark et al., 1988; Ma et al., 1999; Tolay et al., 2001).

Although maize is considered a graminaceous species with low phytosiderophore release (Kawai et al., 1988; Römheld and Marschner, 1990), little is known about other determinants contributing its tolerance to Fe deficiency-induced chlorosis. Studies in different species suggest that such additional determinants may relate to the internal translocation and re-allocation of Fe (Yokosho et al., 2009; Lee et al., 2009; Waters et al., 2009; Shi et al., 2012). Moreover, other nutritional elements have been shown to negatively interfere with Fe acquisition and allocation in plants. In particular, elevated concentrations of heavy metals such as Cd, Mn, Ni, or Zn compete with Fe in different transport processes and partially suppress Fe deficiency responses (Römheld and Marschner, 1986b; Meda et al., 2007; Leskova et al., 2017). Other studies have shown that elevated P supplies decrease the availability and mobility of Fe in soils as well as in plants (DeKock et al., 1979; Zheng et al., 2009) and provoke Fe deficiency-induced chlorosis (Ajakaiye, 1979). Since the first report on the negative impact of increasing P supply on Fe transport and accumulation in bean plants (Rediske and Biddulph, 1953), a series of studies has investigated mechanisms responsible for the negative interference of P on Fe nutrition. Using radioactively labeled ^59^Fe, Kashirad et al. (1973) showed that the presence of phosphate inhibits Fe translocation to the shoots but causes Fe accumulation in the root, presumably due to the precipitation of Fe-phosphates. Indeed, energy dispersive spectroscopy confirmed the existence of ferric phosphate precipitates besides ferric hydroxides on the root surface (Snowden and Wheeler, 1995). Additionally, Zhang et al. (1999) showed that Fe-hydroxides at the root surface can provide adsorption sites for phosphate and thereby build up a phosphate reservoir. Apoplastic Fe is also generated in leaves, where it may contribute to the chlorosis paradox in Fe-deficient plants, i.e., the development of chlorosis symptoms despite a non-critical concentration of leaf Fe (Nikolic and Römheld, 2003). Although there is much less information about the precipitated forms of Fe in the leaf apoplast, recent Perl’s staining approaches in Arabidopsis have shown that more Fe precipitated in the leaf apoplast of ferritin-defective than of wild-type plants, indicating that Fe and P deposition in ferritin proteins created a sink for leaf Fe in the plastids and reduced Fe precipitation in the leaf apoplast (Roschzttardtz et al., 2013). Moreover, the soluble Fe pool in shoots of rice decreased under supply >300 μM P in Fe-sufficient as well as in Fe-deficient plants (Zheng et al., 2009), suggesting that the antagonistic effect of the two elements may also take place in shoots. To date, it is still unclear which physiological processes underlie P-induced Fe deficiency and whether these processes determine the genotypic variability in the sensitivity of Fe nutrition to P.

The poor knowledge on genes governing genotypic variability in Fe efficiency in maize has motivated a series of forward genetic approaches in which Fe efficiency-related traits were assessed in the intermated maize B73 × Mo17 (IBM) population. The IBM population is considered a powerful resource for the analysis of quantitative traits as well as a community standard for genetic mapping and has been successfully used for the identification of QTLs for grain Fe concentrations or Fe efficiency-related traits in maize (Lung’aho et al., 2011; Urbany et al., 2013; Benke et al., 2014). Within this population of 85 tested recombinant inbred lines, we observed that the two parental lines were among the most severely contrasting lines with regard to traits related to Fe deficiency-induced leaf chlorosis (Benke et al., 2014). Interestingly, this contrast was even greater when plants were cultivated under adequate Fe supply and Mo17 consistently suffered from Fe deficiency-induced chlorosis. In an earlier study B73 and Mo17 were assessed for growth performance in P-limited soil, and interestingly, there Mo17 turned out being P efficient, whereas B73 was P inefficient (Kaeppler et al., 2000). Pulling both studies together suggested that B73 and Mo17 may be ideally suited to investigate which physiological processes are responsible for the negative interference of P on Fe nutrition in maize.

In a series of hydroponic experiments, we grew the two genotypes B73 and Mo17 at different Fe:P supply ratios to determine biomass, chlorophyll and mineral element concentrations in shoots and roots as well as Fe and P translocation rates. We then investigated phytosiderophore release and different root traits relevant for Fe–P interactions in the root apoplast. Based on the finding that P supply inactivated more Fe in defined cell wall fractions of roots of the susceptible maize genotype, we assessed the relation between P and Fe in the root cell wall in 85 inbred lines from the tolerant and the susceptible parental line. This combinatorial approach indicated that P-dependent Fe inactivation in the root apoplast is a critical determinant for genotypic differences in the tolerance to Fe deficiency-induced chlorosis.

## Materials and Methods

### Plant Culture

Seeds of maize (*Zea mays* cv. B73 and Mo17) were germinated on filter paper after sterilization in 70% ethanol for 5 min and 15% H_2_O_2_ for 10 min. After 4–5 days, the endosperm was removed and seedlings were transferred in 5-L pots containing nutrient solution of the following composition (in mM): Ca(NO_3_)_2_ 2.0, K_2_SO_4_ 0.7, MgSO_4_ 0.5, KCl 0.1, KH_2_PO_4_ 0.1, Fe(III)EDTA 0.1; (in μM): H_3_BO_3_ 1.0, MnSO_4_ 0.5, ZnSO_4_ 0.5, CuSO_4_ 0.2, (NH_4_)_6_Mo_7_O_24_ 0.01. After one week of preculture, plants were continued to grow on four different Fe and P treatments: 0 μM FeEDTA and 0 μM P (Fe0P0), 0 μM FeEDTA and 100 μM P (Fe0P100), 100 μM FeEDTA and 0 μM P (Fe100P0), 100 μM FeEDTA and 100 μM P (Fe100P100). Alternatively, seedlings were transferred directly to 10, 30, 70, or 200 μM FeEDTA, each at a P supply of 10, 50, or 200 μM. After 17 days, five plants were harvested from different pots, and each plant was handled as one biological replicate. The aerated nutrient solution was renewed every 2 days. During this period, the nutrient solution pH rose from 5.0 to approximately 6.5. P was supplied as KH_2_PO_4_, and the P-deficient solution was balanced by the addition of K_2_SO_4_. Plants were grown in a growth chamber with a 16/8 h light/dark and 25/22°C temperature regime at 60% humidity. The light intensity was approx. 300 μmol photons m^-2^s^-1^. Shoots and roots were frozen in liquid N_2_ and stored at -80°C. For elemental analysis plant samples were dried at 65°C.

### Plant Analysis

The chlorophyll index in young expanded leaves was measured by a SPAD meter 520 (Minolta, Osaka, Japan). Chlorophyll was extracted from shoot samples in N, N’-dimethyl formamide (Merck) after 24 h incubation at 4°C (Porra et al., 1989). Aliquots from shoot samples were ground and fractionated into a water-soluble and water-insoluble fraction, representing readily available nutrient pools (Shi et al., 2012). Solutions were then digested for mineral element analysis. Dried shoot and root samples were ground, wet digested in HNO_3_ and Fe and P concentrations were determined by ICP-OES (Elan 6000; PerkinElmer). Fe and P concentrations in fractionated samples, in xylem sap, or cell wall fractions were determined by high-resolution ICP-MS (Thermo Element2, Bremen, Germany). Apoplastic root Fe was removed as described in von Wirén et al. (1995) and measured according to Bienfait et al. (1985).

Xylem sap was collected as described by Meda et al. (2007). Stems were cut 2–3 cm above the base and silicon tubes were placed on top of the hypocotyl. The first 100 μl of the xylem bleeding sap were discarded before the sap was collected for a period of 3 h, kept on ice and finally stored at -20°C.

### Cell Wall Fractionation

Frozen root samples were ground and cell wall components were extracted as described in Yang et al. (2011) and Zhu et al. (2012a). First, roots were homogenized with a mortar and pestle in 75% (v/v) ice-cold ethanol for 20 min. After centrifugation at 1000 *g* for 10 min, pellets were washed in acetone:methanol:chloroform at a ratio of 2:1:1 (v/v). After another centrifugation, pellets were washed in pure methanol, and cell walls were separated by centrifugation at 1000 *g* for 10 min. The pellet was freeze-dried and pectin (PE) was extracted with hot water (100°C) three times for 1 h each. The residue was incubated two times subsequently in 4% (w/v) KOH and 0.02% (w/v) KBH_4_ (potassium borohydride) for 12 h to extract hemicellulose fraction 1 (HC1). Hemicellulose fraction 2 (HC2) was extracted by 24% KOH for 24 h. For each fraction the supernatants were pooled after centrifugation. The final residue was referred to as cellulose (CE). Before digestion, cellulose was washed several times with distilled water to remove salt.

### Root Exudate Collection Under Axenic Conditions

Seeds of Mo17 and B73 were sterilized and grown in glass bottles under axenic conditions according to von Wirén et al. (1995). The nutrient supply was the same as described but plants were supplemented either without (Fe0P100) or with 100 μM Fe-EDTA (Fe100P100). After growth for 13 days, root exudates were collected every 2 days as described in Carvalhais et al. (2011). Root exudates from three collections were pooled. After filtration (2 μm, nitrocellulose), the exudates were concentrated under vacuum and stored at -20°C. DMA concentrations in root exudates were determined by HPLC as described in Shi et al. (2012).

### Morphological and Histological Analyses

Whole root systems were scanned and primary and total root length were determined using WinRhizo Pro (Regent Instruments, Canada). Prior to scanning, 1-cm segments were cut from the basal part of the primary root (2 cm below the stem) of three independent plants and cross-sectioned. Cross-sections of 3 mm were fixed overnight at 4°C in 50 mM cacodylate buffer, pH 7.2, containing 2%(v/v) glutaraldehyde and 2%(v/v) formaldehyde, followed by one wash with buffer and two washes with distilled water. For secondary fixation, samples were transferred to a solution of 1%(w/v) OsO_4_. After 1 h, samples were washed three times with distilled water. Dehydration at 21°C was performed stepwise by increasing the concentration of ethanol as follows: 30, 40, 50, 60, 75, 90, and 2× 100% ethanol, for 20 min each. Samples were infiltrated with Spurr Resin (Plano, Wetzlar, Germany) as follows: 25% overnight, 50% and 75% resin in 100% ethanol for 4 h each, and then 100% resin overnight (v/v). Samples were transferred into embedding molds, incubated there for 3 h in fresh resin, and polymerized at 70°C for 24 h. Histological examinations were carried out as described previously (Marzec et al., 2013). Scanned histological root cross sections (at least six images per treatment) were used to determine diameters and the area of endodermis, cortex, and cortical aerenchyma using the Image J software and the formula π(D/2)^2^. For each replica, five samples were analyzed taking the average of four area measurements.

### RNA Extraction and Quantitative Real-Time PCR (qRT-PCR) Analysis

Total RNA in shoots and roots was extracted with Trizol reagent (Invitrogen, Germany) and reverse transcribed to cDNA. The qPCR analysis was performed using a commercial PCR kit iQ SYBR Green Supermix (Bio-Rad Laboratories, Hercules, CA, United States). The levels of the RNA for each sample were normalized with respect to the housekeeping gene *ZmGAPDH*. Quantifications were expressed relative to B73 plants grown in complete nutrient solution (Fe100P100). The primers used were: ZmGAPDH-F: CGGCATACACAAGCAGCAAC, ZmGAPDH-R: CTGGTTTCTACCGACTTCCTTG (Gu et al., 2013); ferritin1-F: GCTCGTTGGGATTTGATTGTCGGAA, ferritin1-R: AGGTAGGCAACAGCGAACCTAACCA; ferritin2-F: TGGACGGAGCTGAGCTCTGGGT, ferritin2-R: TAAGAGACAATCCTCCACGTAACATCC.

### Statistics

For statistical analysis, two-way ANOVA was performed, and significant differences among treatments were determined using Tukey’s test (*P* < 0.05). Correlations were determined using the Pearson Product–Moment Correlation. Statistical tests were undertaken using SigmaPlot version 11.0 (Systat Software).

## Results

### B73 and Mo17 Show Differential Sensitivity to Fe Deficiency-Induced Chlorosis in the Presence of P

Based on our previous results identifying B73 and Mo17 as contrasting genotypes with regard to their sensitivity to Fe deficiency-induced chlorosis (Benke et al., 2014), we first characterized the growth response and element composition of B73 and Mo17 plants grown hydroponically at 12 different combinations of Fe and P supplies. In general, both maize genotypes grew without visible symptoms and produced most biomass at 200 μM Fe (F4) and 50 or 200 μM P (P2, P3), also confirming that these upper supply levels did not yet cause growth suppression or Fe and P toxicity (**Table 1**, Supplementary Figure 1). Under provision of 10 μM P (P1), varied Fe supply hardly affected biomass and chlorophyll formation. Mounting Fe supply from 10 to 200 μM increased chlorophyll concentrations significantly in Mo17 but only in tendency in B73, although shoot Fe concentrations increased significantly in both lines and resulted in greener leaves in B73 (Supplementary Figure 1). Shoot and root P concentrations were below 3 mg g^-1^ and thus below the critical deficiency level (Marschner, 2012). At 50 and 200 μM P, chlorophyll concentrations at F1 dropped further, showing a negative impact of P on the Fe nutritional status in shoots. Increasing Fe supply led to a recovery from chlorosis, which in B73 set in at lower Fe supplies than in Mo17. The Fe-dependent increase in shoot and root biomass was similar in both lines (**Table 1**). Shoot Fe concentrations in Mo17 mostly remained below the critical deficiency levels of approximately 50 μg g^-1^, except for F4 at P2 and P3, emphasizing the higher susceptibility of Mo17 to Fe deficiency. In contrast, B73 recovered from Fe deficiency-induced chlorosis at P2 already with 30 μM Fe or at P3 with 70 μM Fe (**Table 1**, Supplementary Figure 1). As Fe concentrations in roots were determined after removal of apoplastic Fe, they reflected physiologically relevant levels, which increased with Fe supply to a similar extent in both genotypes. However, P concentrations differed between the two genotypes. While increasing Fe provision decreased P levels in both genotypes, Mo17 accumulated more P in shoots and roots than B73, especially at P2 and P3. Higher P accumulation in Mo17 was in agreement with the higher P efficiency of Mo17 relative to B73 as observed in greenhouse studies (Kaeppler et al., 2000).

**Table 1:** Influence of varied Fe and P nutritional regimes on growth and the nutritional status in two maize inbred lines.

Treatments		Dry weight (g plant^-1^)				Chlorophyll concentration (μg mg^-1^)		Fe concentration (μg g^-1^)				P concentration (μg g^-1^)			
					
		Shoot		Root				Shoot		Root		Shoot		Root	
									
		B73	Mo17	B73	Mo17	B73	Mo17	B73	Mo17	B73	Mo17	B73	Mo17	B73	Mo17
P1	F1	1.15cde	0.81d	0.39abc	0.40n.s	0.56bcd	0.43ef	51efg	31ef	101g	125ef	1917g	2345e	2413cde	1845d
	F2	1.02cde	1.09bcd	0.33bc	0.42	0.61bcd	0.57bcde	82ab	40cde	219cd	146def	1815g	1808e	2055de	1628d
	F3	1.02cde	1.38abcd	0.36abc	0.49	0.83ab	0.59bcde	73abc	37cde	179de	165cde	1743gh	1413e	1478e	1360d
	F4	1.36bcd	1.50abcd	0.51ab	0.49	0.96ab	0.69bc	73abc	48bc	239bc	166cdef	1327h	1767e	1190e	1520d
P2	F1	0.96de	1.10bcd	0.26cd	0.31	0.15d	0.33fg	40fg	33def	112fg	103f	5605b	5975c	4915a	5700c
	F2	1.25bcd	1.49abcd	0.27cd	0.42	0.73abc	0.55cde	61cde	44c	168e	156def	4290d	5203cd	3463abcd	4420c
	F3	1.55bc	1.53abcd	0.38abc	0.40	0.96ab	0.64bcd	70bcd	42c	206cde	169cde	4218de	4863cd	3293abcd	4210c
	F4	2.17a	1.83ab	0.53a	0.44	0.99ab	0.88a	81ab	54ab	203cde	193cd	3470f	4620d	2665bcde	4143c
P3	F1	0.62e	0.93cd	0.14d	0.25	0.25d	0.23g	33g	29f	158ef	204cd	6643a	10715a	4385abc	9250a
	F2	1.49bcd	1.63abc	0.36abc	0.42	0.31cd	0.49def	56def	38cde	159ef	218c	5083c	8138b	4330abc	7670ab
	F3	1.26bcd	1.40abcd	0.31cd	0.35	1.05a	0.58bcde	87a	41cd	283ab	283b	5420bc	7163b	4428ab	7548b
	F4	1.82ab	1.90a	0.39abc	0.40	1.12a	0.76ab	78abc	55a	290a	348a	3838ef	7568b	3923abc	8390ab
PxFe^∗^		+		+		+	+	+	+	+	+	+	+	+	+


*Dry weights and concentrations of leaf chlorophyll and shoot and root Fe or P in B73 and Mo17 plants. Plants were grown for 17 days in nutrient solution supplemented with 10, 50, or 200 μM P (P1–P3) and with 10, 30, 70, or 200 μM Fe (F1–F4) at all 12 combinations. Values represent mean values and different letters indicate significant differences at *p* < 0.05 (n.s denotes no significance), *n* = 5. ^∗^ describes the interaction between P and Fe, + denotes a statistically significant interaction.*

**FIGURE 1:**
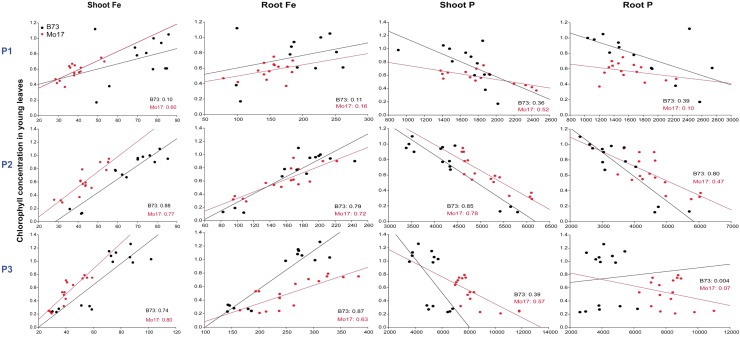
Influence of varied Fe and P nutritional regimes on chlorophyll formation in the maize genotypes B73 and Mo17. Graphs show correlations between chlorophyll (in mg g^-1^) and Fe or P concentrations (in μg g^-1^) in shoots or roots of either B73 or Mo17 plants at each P supply level. Coefficients of correlation (*r*^2^) are indicated for either genotype within the plots. Plants were grown for 17 days in nutrient solution supplemented with 10, 50, or 200 μM P (P1–P3) and with 10, 30, 70, or 200 μM Fe (F1–F4) in all 12 combinations.

We then conducted a series of correlation analyses to look for Fe and P interactions in roots or shoots that relate to the different leaf chlorophyll levels in Mo17 and B73 and may provide a hint whether susceptibility of Mo17 to Fe deficiency is caused by shoot or root factors. For this purpose, we analyzed young leaves, which are most sensitive to Fe deficiency-induced chlorosis. In leaves of both genotypes, chlorophyll concentrations correlated closely with those of Fe, which also held true at highest P supply (**Figure 1**). Thus, despite higher P levels in Mo17, Fe availability for chlorophyll biosynthesis was apparently not impaired. With regard to Fe concentrations in roots, however, Mo17 showed weaker correlation with leaf chlorophyll than B73 when P supply was highest (P3). The flat slope of Mo17 suggested that this genotype requires more root Fe to restore chlorophyll levels in leaves. Thus, this analysis revealed that the availability and conversion of leaf Fe into chlorophyll was similarly efficient in both lines, and suggested that Mo17 requires more root Fe than B73 to achieve adequate chlorophyll levels in the presence of high P.

### Impact of P Supply on Growth and Nutrient Partitioning

**FIGURE 2:**
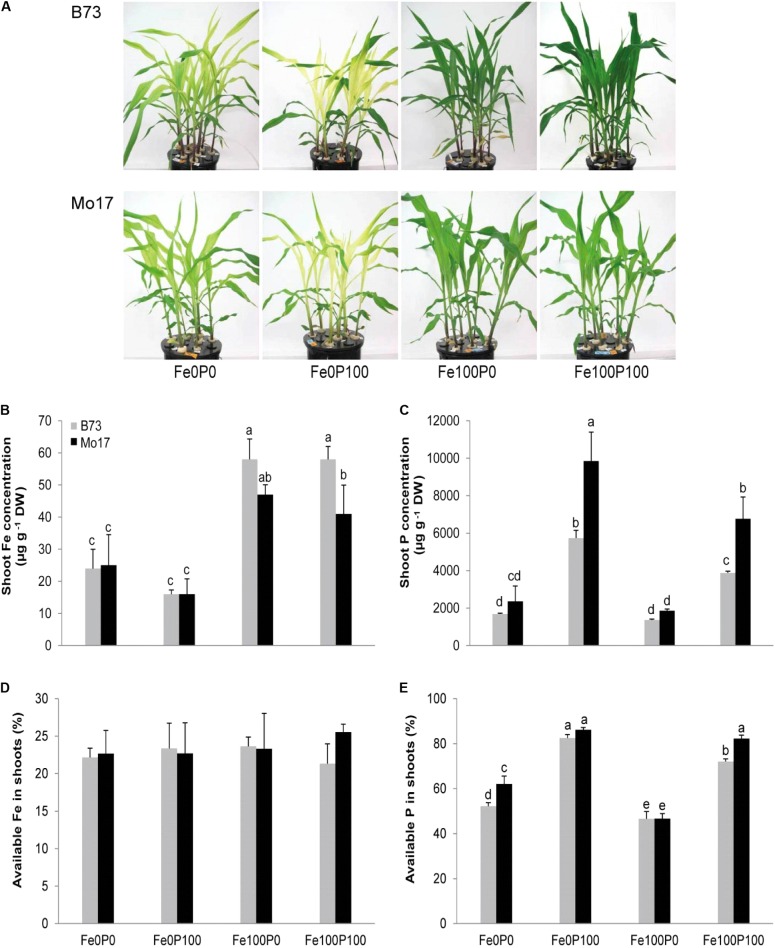
Shoot growth and nutrient concentrations as affected by Fe and P nutritional regimes in B73 and Mo17. **(A)** Shoot phenotype and concentrations of **(B)** Fe, **(C)** P, **(D)**, and proportion of available Fe or **(E)** available P in shoots of B73 and Mo17. Plants were grown for 15 days in nutrient solution supplemented without Fe and P (Fe0P0), without Fe and with 100 μM P (Fe0P100), with 100 μM Fe and without P (Fe100P0) or with 100 μM Fe and 100 μM P (Fe100P100). Fe and P treatments were initiated after one week of preculture on full nutrient solution. Bars indicate mean values ± *SD*, *n* = 5. Different letters denote significant differences at *p* < 0.05.

For a more detailed analysis of Fe and P interactions, we cultivated the two genotypes at four different Fe:P ratios. To avoid toxic effects of P *per se* rather than via P–Fe interactions, P supply was set to 100 μM, which is still among the lowest concentrations commonly used in standard full nutrient solutions (Wang et al., 2002; Stefanovic et al., 2011; Zhu et al., 2012a). In the absence of Fe (Fe0P0, Fe0P100), both maize genotypes developed typical symptoms of Fe deficiency-induced chlorosis (**Figure 2A**). Leaf chlorosis in both lines was moderate in the absence of P and became more severe at adequate P supply (**Table 2**). This severe chlorosis in Fe0P100 plants was not a consequence of Fe dilution in larger shoot biomass, as shoot biomass was not considerably affected. When Fe was adequately supplied, leaves re-greened and SPAD values strongly increased (**Table 2**). However, under concomitant P supply only B73 plants fully recovered from chlorosis and increased shoot dry weight, whereas in Mo17 chlorophyll index and shoot growth remained lower (**Figure 2A** and **Table 2**). Thus, the presence of 100 μM P suppressed shoot growth and chlorophyll formation despite adequate Fe supply only in Mo17, confirming the enhanced sensitivity of its Fe nutrition to external P supply.

**Table 2:** Influence of varied Fe and P nutritional regimes on growth and morphological root traits in two maize inbred lines.

Treatments	Genotype	SPAD	SDW (g plant^-1^)	RDW (g plant^-1^)	TRL (m plant^-1^)	PRL (m plant^-1^)
Fe0P0	B73	7.2 d	1.11 bc	0.58 ab	22.5 bc	67.3 ab
	Mo17	12.8 d	0.93 c	0.40 c	19.4 c	37.2 d
Fe0P100	B73	1.3 e	1.25 b	0.39 cd	10.0 c	46.6 cd
	Mo17	2.4 e	0.89 c	0.26 d	5.3 c	33.9 d
Fe100P0	B73	37.9 b	1.44 b	0.71 a	43.8 b	75.1 a
	Mo17	29.6 b	1.40 b	0.53 bc	23.5 bc	59.2 bc
Fe100P100	B73	44 a	2.07 a	0.69 a	75.6 a	72.8 ab
	Mo17	30.7 c	1.14 bc	0.30 d	24.0 bc	62.3 ab


*Chlorophyll index (SPAD), dry weights of shoots (SDW), and roots (RDW) as well as total root length (TRL) and primary root length (PRL) in B73 and Mo17 plants. Plants were grown for 15 days in nutrient solution supplemented without Fe and P (Fe0P0), without Fe and with 100 μM P (Fe0P100), with 100 μM Fe and without P (Fe100P0) or with 100 μM Fe and 100 μM P (Fe100P100). Fe and P treatments were initiated after one week of preculture on full nutrient solution. Bars indicate mean values, *n* = 5. Different letters denote significant differences at *p* < 0.05.*

In close agreement with the SPAD readings, Fe concentrations in shoots of both genotypes were far below critical deficiency levels when plants were grown without Fe supply (**Figure 2B**). In Mo17, however, shoot Fe concentrations remained below 50 μg g^-1^ even under supply of 100 μM Fe and slightly decreased when P was co-supplied. At the same time, Mo17 responded to P supply with higher P accumulation in shoots at either Fe supply level (**Figure 2C**). In order to investigate whether P supply inactivated Fe in shoots and thereby caused more severe leaf chlorosis, we determined the relative amount of water-soluble Fe and P, representing the proportion of the physiologically active Fe. However, the proportion of the readily available Fe pool remained highly constant irrespective of genotype or soluble shoot P levels (**Figures 2D,E**). Thus, neither total P nor soluble P pools had an obvious impact on soluble Fe pools in leaves, implying that antagonistic interactions between Fe and P in shoots were of minor importance for the higher chlorosis susceptibility of Mo17 to P.

**FIGURE 3:**
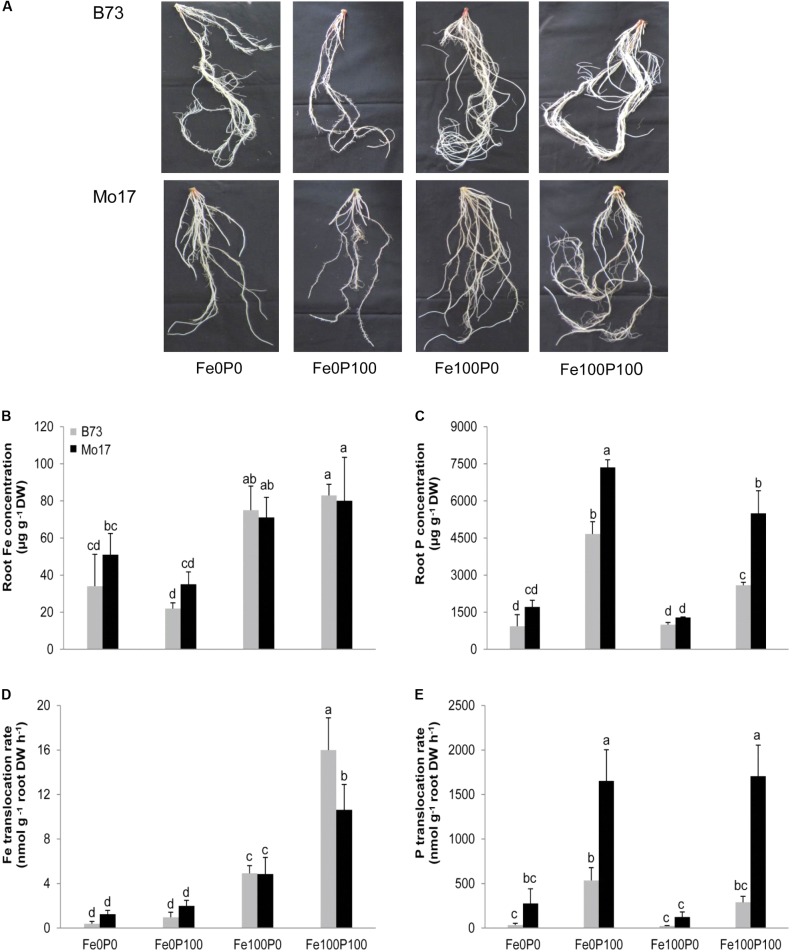
Root growth and physiological root traits as affected by Fe and P nutritional regimes in B73 and Mo17. **(A)** Root anatomy and root concentrations of **(B)** Fe, **(C)** P, or root-to-shoot translocation rates of **(D)** Fe and **(E)** P as determined by collecting xylem sap. Plants were grown for 15 days in nutrient solution supplemented without Fe and P (Fe0P0), without Fe and with 100 μM P (Fe0P100), with 100 μM Fe and without P (Fe100P0) or with 100 μM Fe and 100 μM P (Fe100P100). Fe and P treatments were initiated after one week of preculture on full nutrient solution. Bars indicate means ± *SD*, *n* = 5. Different letters denote significant differences at *p* < 0.05.

A closer look at root traits confirmed the adverse effect of P supply in the two maize genotypes (**Figure 3A**). Under adequate Fe supply, root dry weight of B73 was high irrespective of the level of P supply, whereas in Mo17 root dry weight decreased significantly in the presence of P (**Table 2**). In agreement with studies in Arabidopsis (Gruber et al., 2013), measurements of total root length indicated that Fe deficiency suppressed lateral root formation, which recovered under Fe or Fe plus P supply in B73 but to a much lower extent in Mo17 (**Table 2**). By contrast, in the presence of Fe and P primary root length varied only little between the two genotypes, suggesting that the genotypic difference in total root length under adequate Fe supply was mainly attributed to lateral root extension. Thus, the adverse effect of P supply on shoot growth in Fe-sufficient Mo17 plants was largely reflected in root growth and lateral root development.

Interestingly, the poorer root growth of Mo17 at Fe100P100 was not accompanied by lower Fe concentrations in roots, which were determined here after removal of apoplastic Fe. As expected, root Fe concentrations remained above critical deficiency levels under adequate Fe supply but did not differ significantly between the two genotypes (**Figure 3B**). Like in shoots, P concentrations in roots differed hardly between both genotypes when plants were cultivated under P deficiency, while in the presence of 100 μM P Mo17 accumulated significantly more P in shoots and roots than B73 (**Figures 2C**, **3C**). We therefore assessed whether P suppressed the translocation of Fe from roots to shoots and collected xylem sap for elemental analysis. In fact, Fe translocation rates differed significantly between genotypes only under Fe and concomitant P supply, when Mo17 translocated 34% less Fe to the shoots than B73 (**Figure 3D**). In turn, Mo17 showed much higher translocation rates of P in the xylem (**Figure 3E**), which most likely contributed to the genotypic differences found for P accumulation in the shoots. In conclusion, this series of analyses indicated that the process or factor causing suboptimal Fe provision of Mo17 shoots in the presence of P is located in the roots.

### Phytosiderophore Release by B73 and Mo17

**FIGURE 4:**
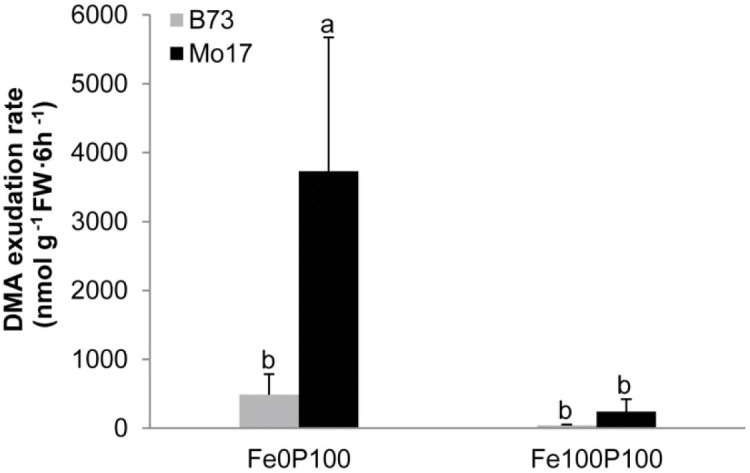
Iron deficiency-induced phytosiderophore release by B73 and Mo17. Plants were grown in nutrient solution containing 100 μM P and 100 μM Fe (Fe100P100) or no Fe (Fe0P100). The whole system was cultivated under axenic conditions. Root exudates were collected three times over a period of 6 h and pooled prior to HPLC analysis of deoxy-mugineic acid (DMA). Bars indicate mean values ± *SD*, *n* = 5. Different letters denote significant differences at *p* < 0.05.

As demonstrated by the continuously chlorotic appearance of the maize mutant *ys1* defective in Fe(III)-phytosiderophore transport (von Wirén et al., 1994), the provision of synthetic Fe(III)-chelates, like Fe(III)-EDTA, to hydroponically grown maize plants requires the involvement of phytosiderophores. To test the hypothesis that Mo17 suffers from lower Fe uptake due to a lower release of phytosiderophores, we measured phytosiderophore release under axenic conditions, because especially in species with poor phytosiderophore release like maize, phytosiderophores are subject to rapid microbial degradation (von Wirén et al., 1994). As expected, P-supplied plants of both genotypes increased the release of deoxymugineic acid (DMA) when precultured under Fe deficiency (**Figure 4**). Unexpectedly, DMA release was severalfold higher in Mo17 than in B73, and the trend for a higher release by Mo17 relative to B73 was also observed when plants were grown in the presence of Fe. We therefore concluded that chlorosis susceptibility in P-supplied Mo17 plants is not due to hampered phytosiderophore biosynthesis or release, and that the higher phytosiderophore release in Mo17 was a consequence of the inadequate Fe nutritional status in Mo17.

### Influence of Fe and P Supply on Ferritin Gene Expression Levels

**FIGURE 5:**
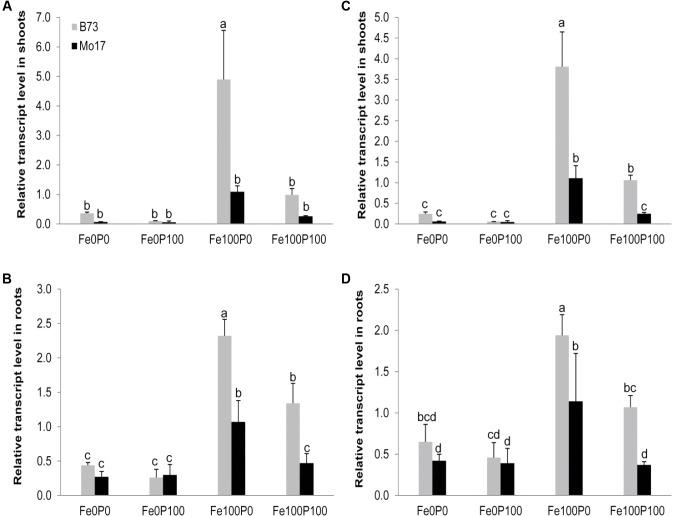
Influence of varied Fe and P nutritional regimes on ferritin gene expression. Relative transcript levels of *ZmFer1* **(A,B)** and *ZmFer2* **(C,D)** in shoots **(A,C)** and roots **(B,D)** of B73 and Mo17 plants grown for 15 days in nutrient solution supplemented without Fe and P (Fe0P0), without Fe and with 100 μM P (Fe0P100), with 100 μM Fe and without P (Fe100P0) or with 100 μM Fe and 100 μM P (Fe100P100). Fe and P treatments were initiated after one week of preculture on full nutrient solution. The housekeeping gene *ZmGAPDH* was used for normalization of mRNA levels, which were set to 1.0 in B73 grown at Fe100P100. Bars indicate mean values ± *SD*, *n* = 4. Different letters denote significant differences at *p* < 0.05.

We then reasoned that a negative interaction between Fe and P could take place inside root cells and analyzed ferritin gene expression levels as read-out for the intracellular Fe nutritional status. As expected from previous studies (Fobis-Loisy et al., 1995), transcript levels of the two major ferritin genes *ZmFER1* and *ZmFER2* were very low under Fe deficiency but strongly increased with Fe supply in shoots as well as in roots (**Figures 5A–D**). Supplementation of P to Fe-sufficient plants has been shown to counteract the Fe-independent induction of ferritin gene transcription by P deficiency (Bournier et al., 2013). Along this line, P supply decreased ferritin transcript levels in roots and shoots of both lines. However, transcript levels of *ZmFER1* and *ZmFER2* were higher in B73 than in Mo17. Presuming that primer matching to the target cDNA for qPCR-mediated amplification was similarly efficient in both lines, this observation may be indicative for a lower intracellular Fe nutritional status in Mo17 shoot and root cells relative to B73. We thus concluded that the genotype-specific interaction between Fe and P took place outside the cytoplasm.

### P Supply Differentially Affects Fe Binding in the Root Apoplast

**FIGURE 6:**
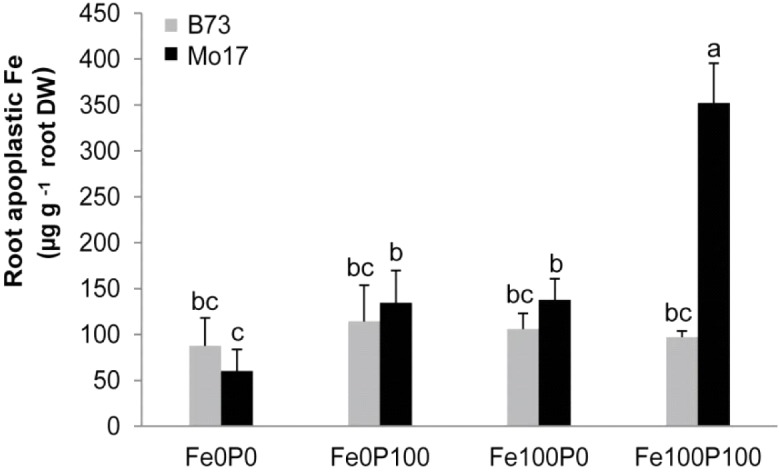
Accumulation of apoplastic Fe under concomitant P supply in the two maize genotypes B73 and Mo17. Plants were grown for 15 days in nutrient solution supplemented without Fe and P (Fe0P0), without Fe and with 100 μM P (Fe0P100), with 100 μM Fe and without P (Fe100P0), or with 100 μM Fe and 100 μM P (Fe100P100). Fe and P treatments were initiated after one week of preculture on full nutrient solution. Bars indicate mean values ± *SD*, *n* = 5. Different letters denote significant differences at *p* < 0.05.

Since the size of the Fe pool in the root apoplast is an important trait in Fe efficiency (Longnecker and Welch, 1990; Zhang et al., 1991), and negative interactions between Fe and P may result from enhanced Fe-phosphate precipitation in the root apoplast (Snowden and Wheeler, 1995), we determined the Fe concentration in the root apoplast of both genotypes. Under Fe deficiency apoplastic root Fe was low (**Figure 6**), mainly resulting from the preculture when Fe was provided to all plants. Interestingly, the apoplastic Fe pool remained low and just increased slightly in Mo17, when Fe was supplied to P-deficient plants (Fe100P0). However, when Fe was co-supplied with P, Fe concentrations in the root apoplast increased by approximately threefold in Mo17 but not in B73 (**Figure 6**). Actually, a similarly drastic difference was found when calculating total Fe contents in the root apoplast (Supplementary Figure 2). Thus, the higher chlorosis susceptibility of Mo17 (**Figure 2**) and lower intracellular Fe nutritional status of Mo17 at equimolar Fe and P supply (**Figure 5**) were supposed to relate to Fe inactivation in the root apoplast.

### Influence of P Supply and Genotype on the Apoplastic Pool Size

**FIGURE 7:**
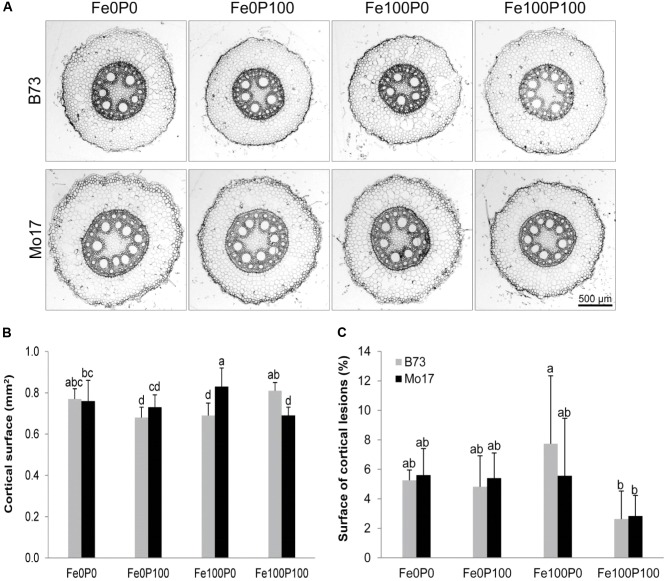
Influence of varied Fe and P nutritional regimes on root anatomy. **(A)** Anatomy of primary roots in cross sections, **(B)** cortical surface, and **(C)** surface of cortical lesions (expressed as proportion of lysed root tissue over the total root cross section). B73 and Mo17 plants were grown for 15 days in nutrient solution supplemented without Fe and P (Fe0P0), without Fe and with 100 μM P (Fe0P100), with 100 μM Fe and without P (Fe100P0), or with 100 μM Fe and 100 μM P (Fe100P100). Fe and P treatments were initiated after one week of preculture on full nutrient solution. Bars indicate mean values ± *SD*, *n* = 5. Different letters denote significant differences at *p* < 0.05.

We hypothesized that the pool size of apoplastic Fe in roots depends primarily on the apoplastic space in outer root cells. As genotypic differences may then derive from a different number of cortical cells or cell layers, we first analyzed root cross sections from the basal part of the primary root for cortical cell area. However, cortical cell layers and the total area of the cortex differed only slightly among the treatments or between the lines, and at Fe100P100 the cortical area of Mo17 was even lower than that of B73 (**Figures 7A,B**). In addition, the total apoplastic space may also be affected by the cortical root senescence, which is an adaptive trait to nutrient deficiency and may promote the remobilization of nutrients from the root cortex (Postma and Lynch, 2011; Schneider et al., 2017). In general, the calculated area for cortical lesions in a cross section ranged between 5 and 6% of the whole cross section (**Figure 7C**). These numbers are in strong agreement with the aerenchyma area of two contrasting maize lines characterized previously (Hu et al., 2014). Under adequate Fe supply, there was indeed a clear trend that P deficiency led to a larger area of cortical lesions. However, in none of the treatments were there significant differences between the two lines. Thus, the higher apoplastic Fe fixation in Mo17 was not primarily related to the inner surface of outer root cells constituting the apoplastic space or to possible differences in radial nutrient transport derived therefrom (Hu et al., 2014).

### P Supply Differentially Affects Fe Binding to Root Hemicelluloses

**FIGURE 8:**
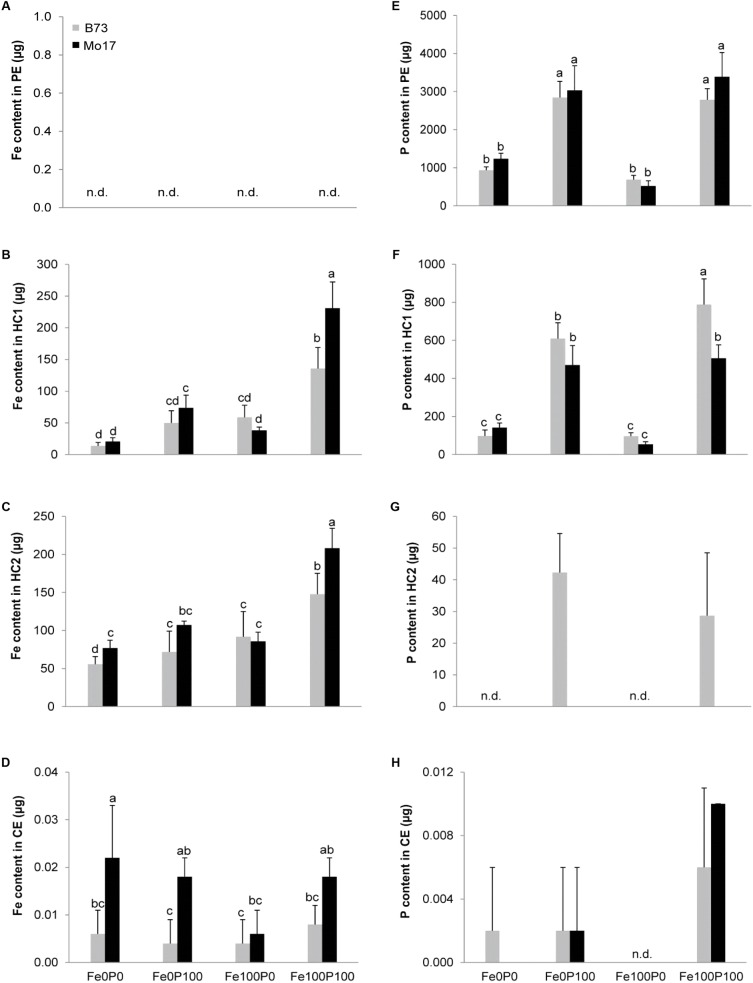
Differential partitioning of Fe and P in cell wall fractions of maize roots. **(A–D)** Fe contents in pectin (PE), hemicellulose fraction-1 (HC1), hemicellulose fraction-2 (HC2), and cellulose (CE); **(E–H)** P contents in pectin (PE), hemicellulose fraction-1 (HC1), hemicellulose fraction-2 (HC2), and cellulose (CE). B73 and Mo17 plants were grown for 15 days in nutrient solution supplemented without Fe and P (Fe0P0), without Fe and with 100 μM P (Fe0P100), with 100 μM Fe, and without P (Fe100P0) or with 100 μM Fe and 100 μM P (Fe100P100). Fe and P treatments were initiated after one week of preculture on full nutrient solution. 1 g of root cell wall material was sequentially extracted for fractionation. Bars indicate mean values ± *SD*, *n* = 5. Different letters denote significant differences at *p* < 0.05.

From a chemical viewpoint, the apoplast is defined by cell wall material which consists of pectin, hemicellulose, and cellulose (Cosgrove, 2005). According to previous studies conducted in Arabidopsis, hemicelluloses in the root apoplast can bind heavy metals, such as Al and Fe, and thereby influence subsequent metal intake (Zhu et al., 2012b; Lei et al., 2014). We therefore grew plants under the same Fe–P regimes as described above and subjected the root material to a sequential extraction procedure which allows collecting cell wall components separately (Yang et al., 2011). While Fe in the pectin fraction was below the detection limit, the large majority of Fe resided in the hemicellulose fractions 1 and 2 (**Figures 8A–C**). Only <1% of the cell wall Fe content remained fixed in the cellulose fraction (**Figure 8D**). This distribution of Fe corresponded roughly to the distribution of Fe observed in different cell wall fractions of Arabidopsis, in which the majority of Fe was also found in hemicellulose fractions (Lei et al., 2014). In these hemicellulose fractions, B73 and Mo17 bound a highly similar amount of Fe, when plants were precultured under Fe deficiency or under Fe supply to P-deficient plants. The significantly larger Fe content found in the cellulose fraction of Mo17 plants made just a negligible contribution to the overall Fe stored in the apoplast (**Figure 8D**). Notably, the most important difference between the two genotypes was found in Fe contents in the hemicellulose fractions-1 and -2 of Fe100P100-supplied plants (**Figures 8B,C**). This difference made up to 150–200 μg Fe g^-1^ root cell wall material and thereby corresponded to more than 80% of the difference in apoplastic root Fe (**Figure 6**) as determined by Fe removal using the classical method of Bienfait et al. (1985).

We then determined the total P content retained in the same cell wall fractions. The large majority of P was fixed in the pectin fraction (**Figure 8E**), where fixed P strongly increased with P supply to plants but was not considerably affected by the maize genotype. The P content in hemicellulose fraction-1 was 5–10 times lower than in the pectin fraction (**Figure 8F**), and here Mo17 contained even significantly less P than B73 in the Fe100P100 treatment. A lower P retention by Mo17 than by B73 was also observed in hemicellulose fraction-2, even though at a lower level and, again, only after continuous P supply (**Figure 8G**). Relative to these three fractions, the amount of P bound to cellulose was negligible (**Figure 8H**). Taken together, this analysis revealed that (i) the majority of Fe was bound in the hemicellulose fractions-1 and -2, while the majority of P was fixed in the pectin fraction, and (ii) the larger amount of Fe found in the hemicellulose fraction of Mo17 in the Fe100P100 treatment contrasted with a smaller amount of P being fixed in the same fraction. Thus, Fe inactivation under elevated P supply was most likely not a direct consequence of Fe–P co-precipitation.

### P-Dependent Chlorosis Susceptibility in the Maize IBM Population

We finally determined whether Fe inactivation in the root hemicellulose fraction is of relevance for chlorosis susceptibility in maize. We examined 85 lines of the maize IBM population including their parents B73 and Mo17 grown at 100 μM P and 10 or 300 μM Fe, i.e., higher Fe supply than before, to promote even more contrasting responses among the inbred lines. Similar to in Benke et al. (2014), we analyzed leaf chlorophyll indices and Fe and P concentrations in different cell wall fractions of the root. Correlations between SPAD values in the youngest, most chlorosis-susceptible leaves with Fe bound in the cell wall were negative and significant (**Table 3**). In plants grown under low Fe supply, SPAD correlated best with Fe fixed in hemicellulose fraction-1 and with P fixed in the pectin fraction. A significant correlation coefficient of 0.4 between hemicellulose-bound Fe and pectin-P further strengthened this interaction in low Fe-supplied plant roots (**Table 3**). In contrast, elevated Fe supply strongly increased the correlation between hemicellulose-bound Fe and cell wall-bound P, which was now most strongly determined by P bound in hemicellulose fraction-1, as would be expected for co-precipitation, followed by pectin-bound P. Taken together, this experiment revealed that hemicellulose-bound Fe relates to pectin-bound P predominantly under low Fe supply, while under elevated Fe supply hemicellulose-bound Fe associated even more tightly with P in the same fraction than with pectin-fixed P.

**Table 3:** Relationships between leaf chlorosis and Fe or P bound in different cell wall fractions of the maize IBM population.

(A) SPAD	Cell wall-Fe	PE-Fe	HC1-Fe	HC2-Fe	CE-Fe
Fe300	-0.382^∗∗^	-0.270^∗∗^	-0.213^∗∗^	-0.146^∗^	-0.269^∗^
Fe10	-0.337^∗∗^	0.183^∗^	-0.406^∗∗^	-0.285^∗∗^	-0.137
**SPAD**	**Cell wall-P**	**PE-P**	**HC1-P**	**HC2-P**	**CE-P**
Fe300	-0.242^∗∗^	-0.335^∗∗^	-0.152^∗^	0.144	0.044
Fe10	-0.398^∗∗^	-0.485^∗∗^	0.015	-0.129	-0.104

**(B) HC1-Fe**	**Cell wall-P**	**PE-P**	**HC1-P**	**HC2-P**	**CE-P**

Fe300	0.752^∗∗^	0.653^∗∗^	0.893^∗∗^	0.385^∗∗^	-0.028
Fe10	0.302^∗∗^	0.400^∗∗^	0.024	0.126	0.096


***(A)** Correlation between SPAD values of youngest leaves with Fe or P concentrations in different cell wall fractions. **(B)** Correlation between hemicellulose 1-bound Fe with P concentrations in different cell wall fractions. 85 lines of the maize IBM population were cultivated hydroponically at two Fe levels, 300 μM Fe (Fe300) or 10 μM Fe (Fe10) before cell wall fractionation and element analysis. PE, pectin fraction; HC1, hemicellulose fraction-1; HC2, hemicellulose fraction-2; CE, cellulose fraction. PE-P, HC1-P, HC2-P, CE-P denote P bound in pectin, hemicellulose fraction-1, hemicellulose fraction-2, or cellulose, respectively. Correlations were calculated according to the Pearson product moment correlation by SigmaPlot version 11.0. ^∗^ and ^∗∗^ indicate significant differences at *p* < 0.05 and *p* < 0.01, respectively.*

## Discussion

Iron efficiency in graminaceous plant species mainly relies on their capacity to increase the synthesis and secretion of phytosiderophores under low Fe availabilities (Kobayashi and Nishizawa, 2012). In graminaceous species with low phytosiderophore synthesis and high sensitivity to Fe deficiency-induced chlorosis like maize (Römheld and Marschner, 1990; Hansen et al., 2006), other factors involved in Fe efficiency and tolerance to Fe deficiency-induced chlorosis may gain in importance. Based on a differential sensitivity of the two widely used maize inbred lines B73 and Mo17 to Fe deficiency-induced chlorosis, we show here that chlorosis tolerance in maize is strongly affected by P supply at physiologically relevant concentrations. These interactions are related to the retention of Fe in the root apoplast, where the level of Fe availability determines Fe–P interactions in different cell wall fractions. Supported by a significant correlation found between chlorophyll index and cell wall-bound Fe and P in a population of 85 maize inbred lines, this study emphasizes the importance of P-dependent Fe retention in the root apoplast as a physiological trait causing genotypic differences in the susceptibility of maize to Fe deficiency-induced chlorosis.

### Iron Inefficiency in Mo17 Relates to Fe–P Interactions in the Root Apoplast

Considering that Fe inactivation can take place in roots as well as in leaves (Nikolic and Römheld, 2003; Zheng et al., 2009), we first addressed this point by comparing the impact of P on Fe deficiency-related traits in the two differentially chlorosis-susceptible lines B73 and Mo17. Correlating leaf chlorophyll with Fe levels in leaves or in roots showed little difference between genotypes, indicating that both genotypes efficiently converted shoot Fe into chlorophyll at any level of P supply (**Table 1** and **Figure 1**). Moreover, the water-soluble fraction of leaf Fe remained unaffected by varied P regimes, indicating that higher P levels in shoots did not decrease Fe availability to a larger extent in Mo17 than in B73, although Mo17 translocated more P to shoots and accumulated P to a larger extent in leaves (**Table 1** and **Figures 2D,E**). These observations supported the view that neither hampered Fe metabolism nor low Fe availability in leaves caused Fe inefficiency in Mo17. Instead, lower Fe translocation rates coinciding with subcritical concentrations of leaf Fe in Mo17 at Fe100P100 (**Figures 2B**, **3D**) strongly indicated that Mo17 is incapable of translocating sufficient amounts of Fe from roots to shoots in the presence of P. Monitoring the translocation of ^59^Fe in maize, Kashirad et al. (1973) observed that root-to-shoot translocation of EDTA-chelated Fe was not considerably affected by external P supply, whereas non-chelated Fe precipitated heavily in the roots and during translocation especially in the presence of P. These observations raised the possibility that Mo17 may be more sensitive than B73 to P-dependent Fe precipitation in roots. However, root Fe concentrations, as determined after the removal of apoplastic Fe, were neither considerably higher nor more affected by external P supply in Mo17 than in B73 (**Table 1** and **Figure 3B**). Only when apoplastic Fe was determined separately, Mo17 revealed a threefold higher fixation of Fe in the root apoplast than B73 (**Figure 6**), corroborating that the major cause for Fe inefficiency in Mo17 related to Fe–P interactions in roots.

With respect to the absolute concentrations of apoplastic Fe generated under our growth conditions, sufficient Fe supply generated a pool size of around 100 μg g^-1^ root DW in B73 (**Figure 6**). This value is in a similar range as that in roots cultivated in membrane bags in soil, where approximately 30–100 μg g^-1^ root DW of apoplastic root Fe was found (Strasser et al., 1999). The corresponding value in Mo17, however, was 350 μg g^-1^ root DW, clearly showing that the genotypic differences largely overrode the difference caused by growing plants in nutrient solution instead of in soil.

One effective mechanism of P-dependent Fe inactivation relates to intracellular Fe storage. In Arabidopsis leaves, energy-dispersive electron microscopy showed that elevated P levels led to an accumulation of phosphate-Fe complexes in vacuoles, whereas under low P supply Fe accumulated inside chloroplasts (Hirsch et al., 2006), going along with the P deficiency-induced upregulation of ferritin (Bournier et al., 2013). Since ferritin also occurs in plastids of maize roots (Lobreaux et al., 1992), we monitored *ZmFER1* and *2* transcript levels, which were consistently downregulated under low Fe supply (**Figures 5A,B**). In Fe-supplied shoots and roots, both genes were upregulated under P deficiency, confirming the dual but independent regulation of ferritins by high Fe and low P (Bournier et al., 2013). However, in both organs, transcript levels were lower in Mo17 than in B73 at either level of P supply (**Figures 5A,B**). Thus, intracellular Fe inactivation by ferritin could not explain P-dependent chlorosis in Mo17 but rather indicated a more deficient Fe nutritional status in roots and shoots of Mo17. Such an interpretation is also in agreement with the observed release of phytosiderophores, which contribute in hydroponic culture to Fe(III) exchange chelation from Fe(III)-EDTA or to Fe(III) mobilization from the root apoplast (Römheld and Marschner, 1990; Zhang et al., 1991). Deoxymugineic acid release from P-supplied roots into a sterile environment was by far larger in Mo17 than in B73 (**Figure 4**), and supported the observation that Mo17 showed consistently higher transcript levels of genes involved in phytosiderophore biosynthesis than B73 even under sufficient Fe supply (Urbany et al., 2013). Considering such high capacity of Mo17 for phytosiderophore-mediated Fe(III) chelation allowed concluding that the negative interaction of P on Fe was downstream of Fe(III) chelation by phytosiderophores but upstream of Fe(III)-phytosiderophore uptake by roots.

### Fe and P Accumulate in Distinct Cell Wall Fractions of the Root

The retention of Fe in the root apoplast has previously been shown to serve as an Fe reservoir during periods of low or lacking Fe supply and consequently been proposed as a critical factor for Fe efficiency in plants irrespective of their strategy for Fe acquisition (Bienfait et al., 1985; Longnecker and Welch, 1990; Zhang et al., 1991; Jin et al., 2007). In the present study, a larger pool of apoplastic Fe in P-sufficient Mo17 roots (**Figure 6**) may have arisen from a higher activity of the constitutive Fe(III)-chelate reductase at the root plasma membrane, which also operates in Fe-sufficient maize roots and generates apoplastic Fe pools by the release of Fe from synthetic Fe(III)-chelates (Bienfait et al., 1985; von Wirén et al., 1993). A sequential chemical extraction of Fe from the root cell wall showed that the bulk of Fe was fixed in the hemicellulose fractions-1 and -2 (**Figures 8B,C**). The differences among treatments and genotypes found by determining Fe contents in this alkaline extraction of cell wall components (**Figure 6**) were highly consistent with the differences in the apoplastic pool size of Fe as determined by ferric Fe reduction and ferrous Fe chelation according to Bienfait et al. (1985) using whole roots. Since Fe binding by the pectin or cellulose fraction was negligible (**Figures 8A,D**), hemicelluloses represent the major cell wall component being responsible for Fe retention in the root apoplast of maize. This is in close agreement with the analysis of Al and Fe pools in Arabidopsis roots that were also fixed in the hemicellulose fraction (Yang et al., 2011; Zhu et al., 2012b; Lei et al., 2014).

The largest amount of P, corresponding to approximately 80% of the cell wall P content, was released from the pectin fraction (**Figure 8E**). Thereby, P fixation in the root apoplast mirrored P supply with approximately threefold larger amounts of P accumulating after continuous P supply than under P deficiency. By contrast, apoplastic root Fe did not increase in the Fe100P0 treatment above the level of that from Fe-deficient plants (**Figure 6**), indicating that this amount of Fe originated from preculture and that further increase in the apoplastic Fe pool strongly depended on concomitant P supply, in particular in Mo17. Nevertheless, during extraction the largest Fe pool was not released with the bulk of P, indicating that co-precipitation of Fe with phosphates in the root apoplast was not the major cause for Fe retention in the root apoplast. Instead, P supply indirectly promoted Fe fixation in the cell wall, presumably via a modulated composition of cell wall components. Such an influence of P nutrition on cell wall biosynthesis has recently been reported in maize roots, where P supply enhanced transcript levels of several genes associated with hemicellulose biosynthesis (Yu et al., 2018). It remains to be investigated which of the hemicellulose moieties tend to bind Fe.

### Iron Retention in Root Hemicelluloses as a Determinant for Genotypic Differences in Chlorosis Susceptibility in Maize

Removal and quantification of apoplastic Fe according to the classical method of Bienfait et al. (1985) showed a dramatic difference between Mo17 and B73, which coincided with enhanced susceptibility of Mo17 plants to Fe deficiency-induced chlorosis in the presence of P. Only under concomitant Fe and P supply, Mo17 roots built up a large pool of apoplastic Fe, while the corresponding Fe pool in B73 plants was almost not affected by P supply (**Figure 6**). Since a larger surface of outer root cells may provide a larger storage capacity for apoplastic Fe, we compared the two genotypes with regard to root anatomy in root cross sections. However, neither the root cortical area, which contributes to most of the inner surface of the root apoplast, nor the P deficiency-induced increase in cortical aerenchyma surface (Postma and Lynch, 2011) differed significantly between the two genotypes (**Figure 7**). Thus, there was no evidence for anatomical traits contributing to genotypic differences in Fe retention in the root apoplast. Also morphological differences were unlikely to be causally involved in a differential Fe retention in root apoplast, as under Fe deficiency root dry weight or total root length did not decrease to a larger extent in Mo17 than in B73 (**Table 2**).

As recently shown in Arabidopsis, P supply can have a strong impact on the chemical composition of root cell walls. P deficiency decreased the concentrations of uronic acids in the pectin and of polysaccharides in the hemicellulose fraction, which coincided with a decreased capacity of the cell wall to bind Cd (Zhu et al., 2012a). This suggested that adequate P supply facilitates Cd binding in the pectin and hemicellulose fractions, thereby promoting Cd toxicity. As another study showed that in particular the hemicellulose fraction-1 in Arabidopsis contains more than 70% of the cell wall-bound Fe (Lei et al., 2014), we first examined Fe retention by different cell wall fractions in the two maize genotypes and found >99% of the cell wall-bound Fe in the hemicellulose fractions-1 and -2. Remarkably, P supply significantly increased the Fe content in both of these fractions and did that to a significantly larger extent in Mo17 than in B73 (**Figures 8B,C**). This relation was confirmed in a subsequent analysis of 85 lines from the IBM population, in which hemicellulose-bound Fe in fraction-1 correlated only with pectin-bound P when Fe supply was low (**Table 3**). By contrast, under elevated Fe supply hemicellulose-bound Fe correlated most tightly with hemicellulose-bound P, which is indicative for co-precipitation and most likely a prominent process underlying antagonistic interactions between Fe and P when supply of both elements is high. The significant correlations between chlorophyll index and hemicellulose-bound Fe as well as pectin-bound P in Fe-deficient IBM lines confirmed the relevance of pectin-bound P in Fe retention in the root apoplast and thus in genotypic differences in chlorosis susceptibility of maize grown under low Fe supplies. In light of these results, we conclude that the relatively low capacity for phytosiderophore release in maize (Kawai et al., 1988; Römheld and Marschner, 1990; **Figure 4**) is insufficient to overcome Fe inactivation in the root apoplast. Then, P-dependent Fe retention in the hemicellulose fraction may become a determining factor for Fe provision of the shoot and thus for chlorosis susceptibility. We therefore propose that this trait deserves more attention when screening and breeding for Fe-efficient maize cultivars.

## Author Contributions

NW and BS conceived the project. RS performed most experiments and analyzed the results. MM performed the microscopy. SZ analyzed part of the cell wall fractions and AB analyzed the IBM population. RS and NW wrote the paper with contribution from all authors.

## Conflict of Interest Statement

The authors declare that the research was conducted in the absence of any commercial or financial relationships that could be construed as a potential conflict of interest.
