# Supplementary material for: Iron Retention in Root Hemicelluloses Causes Genotypic Variability in the Tolerance to Iron Deficiency-Induced Chlorosis in Maize
Source: Front Plant Sci. 2018 Apr 26;9:557. doi: 10.3389/fpls.2018.00557 (PMC5932200; doi:10.3389/fpls.2018.00557)
Supplement: Supplementary file 1 [file Image_1.PDF]

Supplementary Material

Iron retention in root hemicelluloses as a factor for the genotypic variability in iron deficiency-induced chlorosis in maize

Rongli Shi[1], Michael Melzer[1], Shaojian Zheng[2], Andreas Benke[3], Benjamin Stich[3], Nicolaus von Wirén[1*]

*** Correspondence:**

Nicolaus von Wirén

email: vonwiren@ipk-gatersleben.de

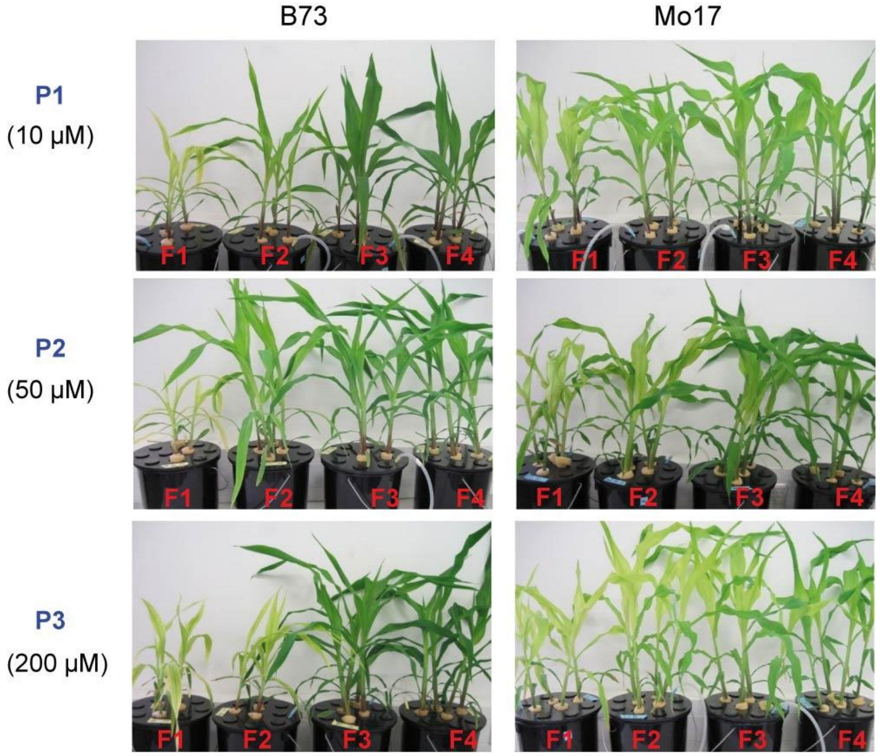

Supplementary Figure 1. Influence of varied Fe and P nutritional regimes on Fe deficiency symptoms in the shoot of the two maize genotypes B73 and Mo17. Plants were grown for 17 days in nutrient solution supplemented with 10, 50 or 200 µM P in P1-P3, respectively, and with 10, 30, 70 or 200 µM Fe in F1-F4, respectively, in all 12 combinations.

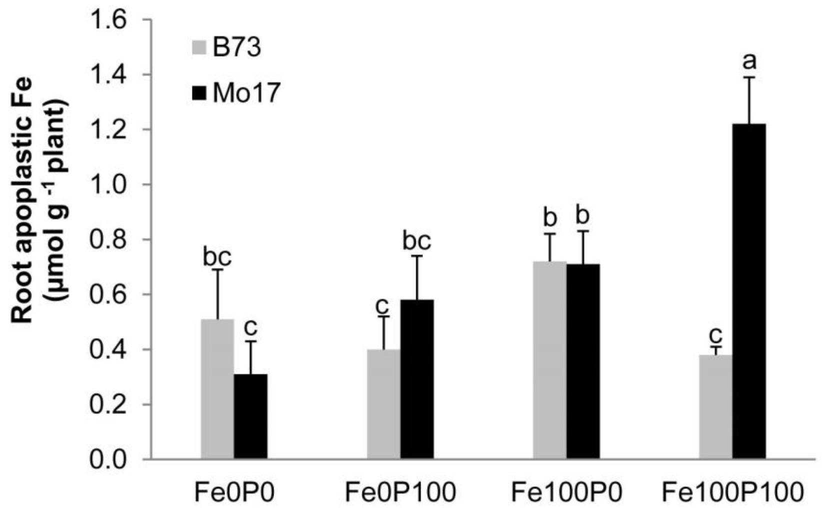

Supplementary Figure 2. Total Fe contents in the root apoplast as affected by Fe and P supply in the two maize cultivars B73 and Mo17. Plants were grown for 15 days in nutrient solution supplemented without Fe and P (Fe0P0), without Fe and with 100 µM P (Fe0P100), with 100 µM Fe and without P (Fe100P0) or with 100 µM Fe and 100 µM P (Fe100P100). Fe and P treatments were initiated after one week of preculture on full nutrient solution. Bars indicate means ± SD, n=5. Different letters denote significant differences at $p<0.05$.
